# 基质固相分散萃取-高效液相色谱法同时测定五味子中5种木脂素类化合物

**DOI:** 10.3724/SP.J.1123.2022.05012

**Published:** 2023-03-08

**Authors:** Xinxin DU, Yinpeng WANG, Wei XIAO, Jingbo ZHU

**Affiliations:** 1.大连工业大学食品学院, 辽宁 大连 116034; 1. School of Food Science and Technology, Dalian Polytechnic University, Dalian 116034, China; 2.中药制药过程新技术国家重点实验室, 江苏 连云港 222001; 2. State Key Laboratory of New-tech for Chinese Medicine Pharmaceutical Process, Lianyungang 222001, China

**Keywords:** 基质固相分散萃取, 高效液相色谱, 木脂素类化合物, 五味子, matrix solid-phase dispersion extraction (MSPD), high performance liquid chromatography (HPLC), lignans, *Schisandra chinensis*

## Abstract

研究建立了基质固相分散萃取-高效液相色谱(MSPD-HPLC)分析五味子中5种木脂素类化合物(五味子醇甲、五味子醇乙、五味子甲素、五味子乙素、五味子丙素)的方法。采用反相C_18_色谱柱进行分离,以0.1%(v/v)甲酸水溶液和乙腈为流动相进行梯度洗脱,在波长250 nm下检测。考察了包括硅胶、酸性氧化铝、中性氧化铝、碱性氧化铝、佛罗里硅土、Diol、XAmide、Xion和C_18_、C_18_-ME、C_18_-G_1_、C_18_-HC等在内的12种吸附剂以及吸附剂的质量、洗脱剂的种类、洗脱剂体积对五味子木脂素类化合物得率的影响。选定Xion作为MSPD-HPLC分析五味子中木脂素类化合物的吸附剂;基于吸附剂Xion的萃取参数优化结果表明:以0.25 g五味子粉末为固定值,Xion(0.75 g)为吸附剂,甲醇(15 mL)为洗脱剂,MSPD对五味子中木脂素类化合物具有较高的得率。建立的五味子中5种木脂素类化合物的分析方法,各目标分析物具有良好的线性关系(相关系数*R*^2^≥0.9999),检出限与定量限分别介于0.0089~0.0294 μg/mL和0.0267~0.0882 μg/mL之间。对五味子木脂素类化合物进行低、中、高3个水平的加标回收试验,平均回收率为92.2%~111.2%,相对标准偏差为0.23%~3.54%。日内和日间精密度均小于3.6%。与超声辅助提取和热回流提取前处理相比,MSPD具有萃取和净化相结合、耗时少、所需溶剂量少的优点,且MSPD-HPLC获得的结果优于经典方法。所建立的方法成功应用于17批五味子中5种木脂素类化合物含量的分析。

## Abstract (en)

The kidney-shaped, red-colord fruit from the plant, *Schisandra chinensis* (Turcz.) Baill, which belongs to the Schisandraceae family, is among the most popular remedies used in traditional Chinese medicine. The English name of the plant is “Chinese magnolia vine”. It has been used in Asia since ancient times to treat a variety of ailments, including chronic cough and dyspnea, frequent urination, diarrhea, and diabetes. This is because of the wide range of bioactive constituents, such as lignans, essential oils, triterpenoids, organic acids, polysaccharides, and sterols. In some cases, these constituents affects the pharmacological efficacy of the plant. Lignans with a dibenzocyclooctadiene-type skeleton are considered to be the major constituents and main bioactive ingredients of *Schisandra chinensis*. However, because of the complex composition of *Schisandra chinensis*, the extraction yields of lignans are low. Thus, it is particularly important to study pretreatment methods used during sample preparation for the quality control of traditional Chinese medicine. Matrix solid-phase dispersion extraction (MSPD) is a comprehensive process involving destruction, extraction, fractionation, and purification. The MSPD method is simple, it requires only a small number of samples and solvents, it does not require any special experimental equipments or instruments, and it can be used to prepare liquid, viscous, semi-solid, solid samples.

In this study, a method combining matrix solid-phase dispersion extraction with high performance liquid chromatography (MSPD-HPLC) was established for the simultaneous determination of five lignans (schisandrol A, schisandrol B, deoxyschizandrin, schizandrin B, and schizandrin C) in *Schisandra chinensis*. The target compounds were separated on a C_18_ column with a gradient elution of 0.1% (v/v) formic acid aqueous solution and acetonitrile as the mobile phases, and detection was performed at a wavelength of 250 nm. First, the effects of 12 adsorbents, including silica gel, acidic alumina, neutral alumina, alkaline alumina, Florisil, Diol, XAmide, Xion, and the inverse adsorbents, C_18_, C_18_-ME, C_18_-G_1_, and C_18_-HC, on the extraction yields of lignans were investigated. Second, effects of the mass of the adsorbent, the type of eluent, and volume of eluent on the extraction yields of lignans were investigated. Xion was chosen as an adsorbent for MSPD-HPLC analysis of lignans from *Schisandra chinensis*. Optimization of the extraction parameters showed that the MSPD method had a high lignan extraction yield with *Schisandra chinensis* powder (0.25 g) as a fixed value, Xion as the adsorbent (0.75 g), and methanol as the elution solvent (15 mL). Analytical methods were developed for five lignans from *Schisandra chinensis* and these methods showed good linearity (correlation coefficients (*R*^2^)≥ 0.9999) for each target analyte. The limits of detection and quantification ranged from 0.0089 to 0.0294 μg/mL and 0.0267 to 0.0882 μg/mL, respectively. Lignans were tested at low, medium, and high levels. The average recovery rates were 92.2% to 111.2%, and the relative standard deviations were 0.23% to 3.54%. Both intra-day and inter-day precisions were less than 3.6%. Compared with hot reflux extraction and ultrasonic extraction methods, MSPD has the advantages of combined extraction and purification, being less time-consuming, and requiring lower solvent volumes. Finally, the optimized method was successfully applied to analyze five lignans from *Schisandra chinensis* samples from 17 cultivation areas.

五味子是木兰科五味子属(*Schisandra chinensis*(Turcz.)Baill)的成熟干燥果实,在《神农本草经》中最初被记录,具有收敛固涩、益气生津、补肾宁心的功效^[1]^。作为中药或保健食品原料,五味子的主要有效成分是木脂素类化合物^[2]^,其含量分析对于以五味子为原料的中药及保健食品的质量分析具有重要意义。

提取、净化等样品前处理过程是植物样本中化学成分分析的关键过程。《中国药典》(2020版)规定,五味子中木脂素的分析采用溶剂多次提取的方法^[3]^,传统的索氏提取^[4]^、热回流^[5]^、超声辅助提取^[6]^等方法已被用于五味子成分分析的前处理,其存在的主要问题是:制备时间较长、消耗大量有机溶剂及清洗、过滤、浓缩和转移等实验步骤繁杂,既不符合绿色化学理念,也会因为繁杂的步骤引入不必要的误差。近年来,植物基源样本分析前处理研究重点在于如何减少有机溶剂消耗、消除额外成分对色谱分析的影响、省略样品转移和预浓缩步骤以及提高萃取效率等。Barker等^[7]^于1989年提出的基质固相分散萃取法(MSPD)是一种简单且廉价的样品前处理方法,其基本原理和过程是将样品与固体吸附剂以一定比例直接混合后机械研磨,形成的样品与吸附剂混合均一的固相介质置于固相萃取管内,以合适的溶剂洗脱,选择性分离出一种或几种化合物后定容,完成集植物细胞破碎、萃取、分馏和纯化于一体的样品前处理过程。

迄今为止,MSPD已被用于化妆品^[8]^、食品成分分析^[9]^、农兽药残留检测^[10]^、中药及其制剂^[11]^、草药中精油^[12]^等的应用中。另外,对五味子中木脂素类化合物的测定分析也有报道,Zhang等^[13]^于2016年首次采用MSPD技术从五味子中提取分析了5种具有二苯并环辛二烯型骨架的木脂素类化合物。Song等^[14]^采用中性氧化铝为介质的MSPD-HPLC实现了五味子中6种木脂素的快速分析。

MSPD应用于诸如中药等复杂体系分析的前处理,吸附剂及合适的洗脱条件选择是关键,吸附剂参与样品的破碎过程,并将分析对象吸附到固相载体上,而有效的洗脱可以确保获得准确的分析结果。常用的吸附剂包括正相吸附剂(如硅胶、氧化铝、佛罗里硅土)、反相吸附剂(以C_18_为基质)和无吸附性吸附剂(如海沙等)^[15]^,这些吸附剂通常表现出较低的选择性。1990年,Alpert提出了亲水相互作用色谱法(HILIC)^[16]^,作为反相色谱法的补充方法,HILIC可以有效保留植物中存在的如糖苷、糖肽、酚类、有机酸等极性化合物^[17]^,而对弱极性化合物的保留较小,应该可以被用于植物中弱极性木脂素类化合物的富集与净化。

本文采用MSPD结合HPLC分析研究建立五味子中5种木脂素类化合物的分析方法。考察包括正相色谱、反相色谱及亲水相互作用色谱等3个类型的12种吸附剂对五味子中木脂素得率的影响,选择合适的萃取材料和萃取条件。将建立的方法与超声辅助提取和热回流提取前处理方法相比较,验证该方法与经典分析方法及其预期目的适应性。

## 1 实验部分

### 1.1 仪器、试剂与材料

AC Chrom S6000型高效液相色谱分析仪(北京华谱新创科技有限公司), 12 mL固相萃取柱(湖北苏林科技有限公司), 600Y型高速多功能粉碎机(永康市铂欧五金制品有限公司), DHG-3A型电热恒温鼓风干燥箱(巩义市予华仪器有限责任公司), KQ-500B型超声波清洗器(巩义市英裕高科仪器厂)。

五味子甲素(schisandrol A,纯度99.5%)、五味子乙素(schisandrol B,纯度99.1%)购自中国食品药品检定研究所。五味子醇甲(deoxyschizandrin)、五味子醇乙(schizandrin B)、五味子丙素(schizandrin C)标准物质由本实验室从五味子萃取物中分离纯化,并通过^1^H-NMR、^13^C-NMR和HPLC-MS进行了鉴定,纯度均在98%以上。硅胶、C_18_、C_18_-ME、C_18_-G_1_、C_18_-HC、Diol、XAmide、Xion均购自北京华谱新创科技有限公司;酸性氧化铝、碱性氧化铝、中性氧化铝购自上海陆都化学试剂厂;色谱级甲醇、乙腈购自天津康科德科技有限公司;分析级甲醇、乙腈、甲酸、丙酮购自天津市科密欧化学试剂有限公司。

本文17批五味子样品经鉴定均为木兰科五味子属的成熟干燥果实(北五味子),于2021年10~12月购自中国不同地区。

### 1.2 标准溶液的配制

准确称取五味子醇甲、五味子醇乙、五味子甲素、五味子乙素、五味子丙素标准品适量,于甲醇中溶解,配制成质量浓度分别为1.34、1.10、1.00、0.98和1.02 mg/mL的混合标准溶液,于4 ℃冰箱冷藏保存备用。根据需要,混合标准工作液由混合标准溶液经甲醇溶液逐级稀释至适当浓度。

### 1.3 样品预处理

五味子药材于65 ℃电热恒温鼓风干燥箱中烘干12 h。用高速多功能粉碎机进行粉碎,过40目标准试验筛,储存在-4 ℃冰箱备用。

### 1.4 样品前处理

#### 1.4.1 MSPD

称取五味子粉末0.25 g,与吸附剂Xion 0.75 g混合,室温条件下充分研磨3~5 min。将研磨后的样品转移至12 mL固相萃取柱空管中,萃取柱上、下两端放置筛板,充分压实;加入15 mL甲醇,使用注射器加压洗脱,收集洗脱液于25 mL容量瓶中,并用甲醇定容至刻度。0.22 μm滤膜过滤后进行HPLC分析。

#### 1.4.2 超声辅助提取法

称取五味子粉末0.25 g,转移至试管中,然后加入15 mL甲醇溶液称重,在功率200 W的条件下超声提取1 h后将样品冷却至室温,再次称重,并用甲醇补足减少的重量。随后,将样品混合物过滤。0.22 μm滤膜过滤后进行HPLC分析。

#### 1.4.3 热回流法

将0.25 g五味子粉末与15 mL甲醇混合于锥形烧瓶中,准确称量,通过热回流法提取1 h,随后冷却至室温,再次称量,并用甲醇补足损失的重量,样品提取物混合均匀后,收集滤液,将所得溶液过0.22 μm滤膜,用于HPLC分析。

### 1.5 色谱条件

色谱柱:Bomex FINDSIL C_18_色谱柱(250 mm×4.6 mm, 5 μm);流动相A:乙腈,流动相B: 0.1%(v/v)甲酸水溶液;柱温:30 ℃;流速:1 mL/min;进样量:20 μL;检测波长:250 nm。梯度洗脱程序:0~10 min, 10%A~50%A; 10~60 min, 50%A~100%A。基于MSPD-HPLC分析五味子样品的色谱图(见图1)。

**图1:**
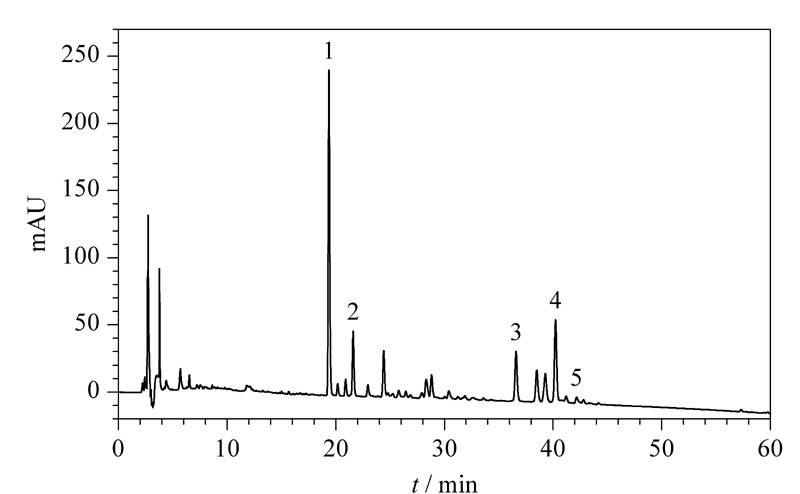
基质固相分散法萃取木脂素类化合物的高效液相色谱图

## 2 结果与讨论

### 2.1 吸附剂的选择

吸附剂的类型及吸附机理决定其选择性,以及样品与吸附剂之间能否产生高吸附能力。本文考察了正相色谱填料(硅胶、佛罗里硅土、酸性氧化铝、中性氧化铝、碱性氧化铝)、HILIC填料(Xion、XAmide、Diol)及反相色谱填料(C_18_-HC、C_18_-G_1_、C_18_、C_18_-ME)等在内的12种吸附剂对五味子木脂素类化合物得率的影响(见图2)。以上12种吸附剂对目标分析物具有相似的得率。反相吸附剂对五味子甲素和五味子乙素表现出较低的得率,这可能是因为C_18_材料的非极性和疏水性吸附机理,而五味子甲素和乙素的极性相对于五味子醇甲和五味子醇乙较弱,使目标分析物的洗脱更难,导致其得率较低^[18]^。如图2数据所示,相比而言HILIC型填料对五味子木脂素类化合物的得率较高。其中Xion的得率略高于其他正相、反相吸附剂,这与其采用独特的两性离子键合技术、对弱极性化合物的保留较弱的特性密切相关^[19]^,适合极性相对较弱的五味子木脂素类化合物富集。

**图2:**
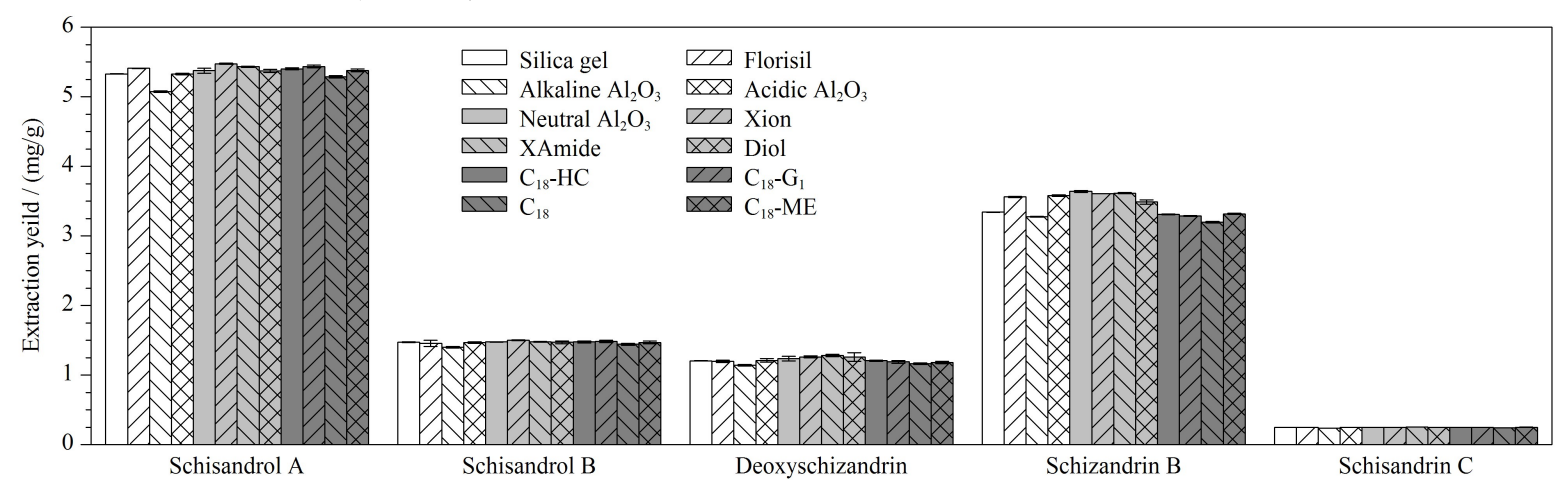
不同吸附剂对目标分析物得率的影响(*n*=3)

### 2.2 吸附剂质量的选择

吸附剂与样品的质量比会影响样品与吸附剂的接触面积和吸附效率。本文以0.25 g五味子粉末为固定值,通过改变吸附剂的质量对五味子木脂素类化合物得率进行评估(见图3)。当Xion的质量从0.125 g上升到0.75 g时,各目标化合物的得率最高。这可能是因为合适的比例会增加样品和吸附剂之间的接触面积,并使样品完全吸附于Xion,使得吸附剂与样品产生更强的分子相互作用^[20]^,从而提高目标分析物的得率。当样品与Xion的质量在0.75 g~1.25 g范围内时,得率呈下降趋势。这可能是由于随着吸附剂量的增大,所选的15 mL溶剂并未使目标分析物从MSPD柱中完全洗脱,从而使得率呈现逐渐降低的趋势。因此,本实验选择吸附剂的质量为0.75 g。

**图3:**
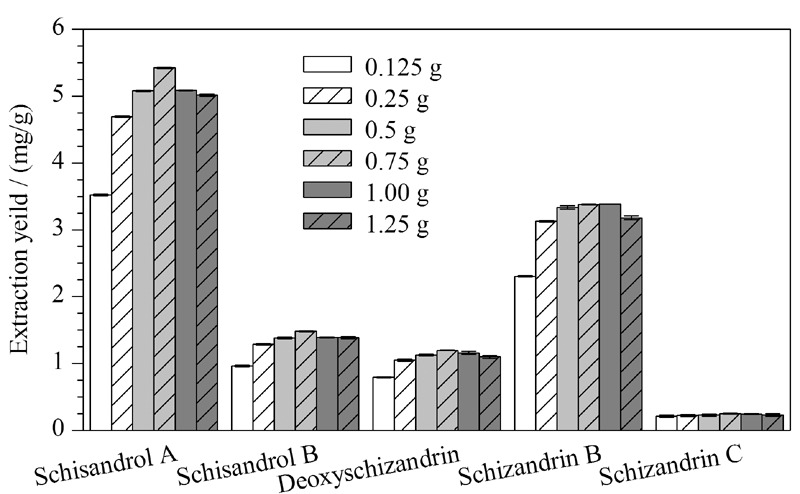
吸附剂质量对目标分析物得率的影响(*n*=3)

### 2.3 洗脱剂的选择

洗脱剂对MSPD过程中目标分析物的有效洗脱有显著影响。以Xion填料作为吸附剂,首先以甲醇、乙醇、丙酮、乙腈为洗脱剂对五味子木脂素类化合物得率进行评估,结果见图4。4种溶剂对五味子木脂素类化合物均具有良好的洗脱,相比而言,乙腈对五味子乙素有较高的得率,而甲醇对于五味子醇甲、五味子醇乙、五味子甲素的得率更高,因此选择甲醇作为洗脱剂进行下一步试验。

**图4:**
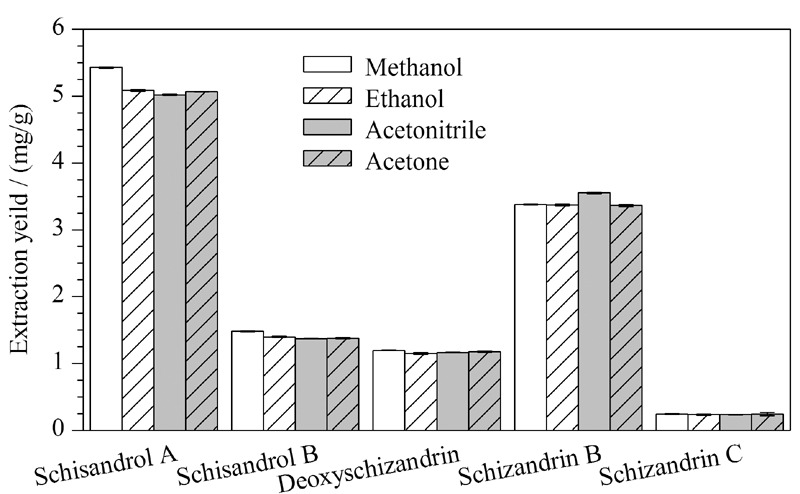
不同洗脱剂对目标分析物得率的影响(*n*=3)

接下来研究了在相同洗脱体积(15 mL)下,不同体积分数的甲醇水溶液作为洗脱剂对五味子中木脂素类化合物得率的影响(见图5)。20%甲醇水溶液作为洗脱剂时,未检出五味子乙素和五味子丙素,40%甲醇水溶液作为洗脱剂时,未检测出五味子丙素,随着甲醇水溶液的体积分数由20%增加到80%,木脂素的得率也逐渐增加;而100%甲醇溶液与80%甲醇水溶液作为洗脱剂时得率变化不显著。实验过程中发现,洗脱剂中水的存在会导致溶剂的黏度变大,洗脱时间也相应增加^[21]^,洗脱剂不易通过固相萃取柱。当洗脱剂中甲醇含量保持在较高水平时,得率显著增强且洗脱时间缩短,因此,选择100%甲醇溶液作为洗脱剂。

**图5:**
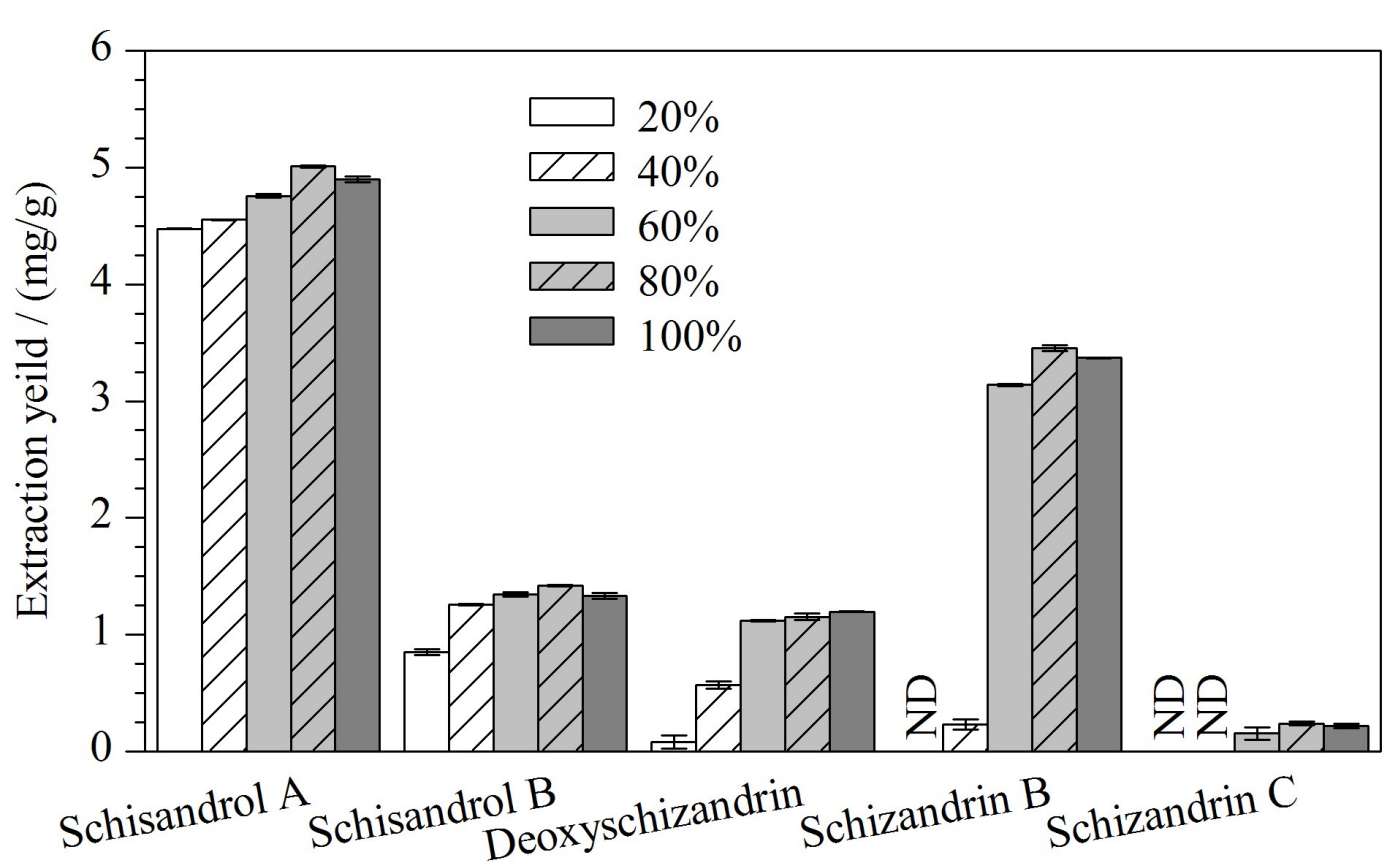
甲醇体积分数对目标分析物得率的影响(*n*=3)

### 2.4 洗脱剂体积的选择

不同洗脱剂体积对目标化合物具有不同的洗脱效果。分别考察了5、10、15、20和25 mL甲醇对五味子木脂素类化合物得率的影响(见图6)。当洗脱剂体积为15 mL时得率达到最高,而洗脱剂用量由15增加到25 mL时,得率再无明显提高。实验选择洗脱剂体积为15 mL。

**图6:**
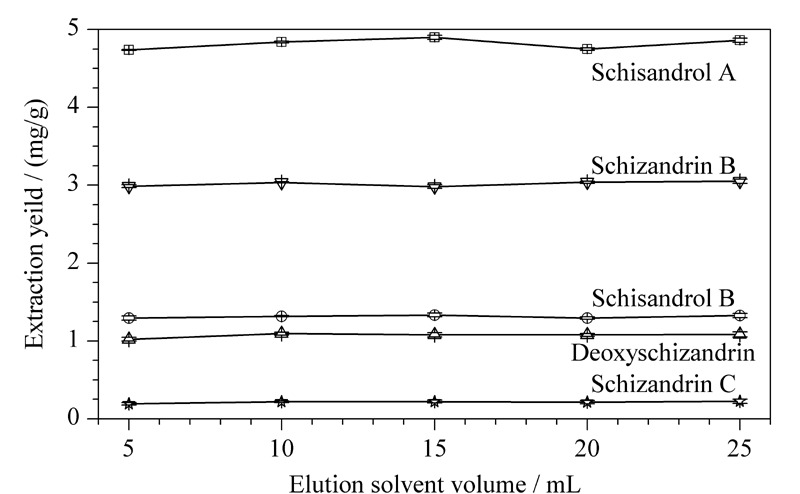
洗脱剂的体积对目标分析物得率的影响(*n*=3)

### 2.5 方法学验证

#### 2.5.1 线性关系、检出限和定量限

按1.2节方法配制混合标准工作液,并分别进行测定。以各分析物峰面积*Y*作为纵坐标,质量浓度*X* (mg/mL)作为横坐标,绘制5种木脂素类化合物的标准曲线,以信噪比(*S/N*)=3和*S/N*=10分别计算分析方法的检出限(LOD)和定量限(LOQ)。5种木脂素类化合物在各自的线性范围内具有良好的线性关系(相关系数*R*^2^≥0.9999), LOD与LOQ分别介于0.0089~0.0294 μg/mL和0.0267~0.0882 μg/mL之间(见表1)。

**表1:** 5种木脂素类化合物的线性范围、线性方程、相关系数、检出限和定量限

Compound	Linear range/(μg/mL)	Linear equation	R^2^	LOD/(μg/mL)	LOQ/(μg/mL)	
Schisandrol A	0.429-268	Y=3.99×10^7^X+4.34×10^4^	0.9999	0.0098	0.0294	
Schisandrol B	0.326-204	Y=3.20×10^7^X+1.94×10^4^	0.9999	0.0109	0.0327	
Deoxyschizandrin	0.314-196	Y=3.92×10^7^X+2.34×10^4^	0.9999	0.0294	0.0882	
Schizandrin B	0.320-200	Y=4.60×10^7^X+5.07×10^4^	0.9999	0.0089	0.0267	
Schizandrin C	0.352-220	Y=2.91×10^7^X+1.78×10^4^	0.9999	0.0211	0.0633	

Y: peak area; X: mass concentration, mg/mL.

#### 2.5.2 精密度和稳定性

按照1.4.1节方法制备供试品,连续3 d分别对样品进样6次,测定该方法的日内精密度和日间精密度。结果表明,5种木脂素类化合物的日内精密度在0.41%~0.95%之间,日间精密度在2.4%~3.6%之间。

将供试品溶液置于室温下,分别在制备后的0、2、4、6、12、24 h后进样测定,结果显示5种木脂素类化合物峰面积的RSD分别为0.21%、0.16%、0.42%、4.2%、0.78%,表明五味子供试品溶液在24 h内基本稳定。

#### 2.5.3 回收率

称取已知含量的五味子样品0.25 g,按照1.4.1节方法对样品进行处理后,进行HPLC测定,记录各分析物的峰面积,根据木脂素类化合物在本实验所测定的浓度,添加一定量(低、中、高水平)的混合标准溶液至实际样品中,测得方法的回收率在92.2%~111.2%之间,结果见表2。

**表2:** 5种木脂素类化合物的回收率和相对标准偏差(n=6)

Compound	Background/(mg/mL)	Added/(mg/mL)	Found/(mg/mL)	Recovery/%	RSD/%	
Schisandrol A	0.046	0.0300	0.074	94.7	0.32	
		0.0600	0.105	97.5	1.34	
		0.0900	0.146	111.2	0.71	
Schisandrol B	0.013	0.0085	0.021	99.6	1.22	
		0.0170	0.030	97.6	1.50	
		0.0225	0.038	110.0	0.64	
Deoxyschizandrin	0.010	0.0070	0.016	92.4	0.23	
		0.0014	0.011	105.6	1.74	
		0.0021	0.012	104.8	0.93	
Schizandrin B	0.027	0.0125	0.039	94.5	0.34	
		0.0250	0.050	92.2	2.12	
		0.0375	0.068	109.1	0.81	
Schizandrin C	0.0019	0.0015	0.003	95.0	0.78	
		0.0030	0.005	99.3	1.60	
		0.0045	0.006	98.4	3.54	

### 2.6 MSPD与经典方法对比

本文方法与热回流法和超声辅助提取法相比较的结果见表3。使用相同的料液比条件进行样品前处理时,MSPD中5种木脂素类化合物的得率高于经典方法,并且MSPD具有萃取时间短、样品量少、溶剂体积小等优点。

**表3:** MSPD与经典方法测定五味子木脂素类化合物得率的比较

Compound	Extraction yields/(mg/g)		
	Hot reflux extraction	Ultrasonic extraction	MSPD
Schisandrol A	4.50	4.77	5.85
Schisandrol B	1.27	1.35	1.63
Deoxyschizandrin	1.02	1.11	1.34
Schizandrin B	3.11	3.15	3.70
Schizandrin C	0.21	0.24	0.32

### 2.7 不同产地五味子样品含量测定

采用建立的方法分析了不同地区五味子中木脂素类化合物的含量,见表4。5种木脂素类化合物在17份样品中均有存在,但不同地区的五味子中木脂素类化合物含量有明显差异。来自黑龙江尚志的五味子,其五味子乙素含量高达7.57 mg/g,辽宁清原的五味子中五味子乙素含量仅为2.48 mg/g。这种显著的差异可能与栽培地理来源、生长气候条件、收获期和贮藏有关。

**表4:** 不同产地17份五味子中木脂素类化合物的含量

Province	Area	Lignan contents/(mg/g)				
		Schisandrol A	Schisandrol B	Deoxyschizandrin	Schizandrin B	Schizandrin C
Liaoning	Dandong	4.96	1.46	0.99	3.95	0.38
	Fengcheng	4.73	1.56	1.00	4.14	0.41
	Kuandian	3.90	1.40	0.85	3.91	0.41
	Huanren	4.82	1.23	1.49	3.70	0.38
	Qingyuan	4.73	1.91	0.56	2.48	0.31
	Tieling	5.05	1.29	1.38	2.80	0.33
Jilin	Ji’an	5.02	2.23	1.04	4.35	0.40
	Antu	4.80	1.35	1.01	3.11	0.31
	Baishan	4.61	1.59	1.08	3.21	0.46
	Jingyu	5.47	1.50	1.26	3.61	0.25
Heilongjiang	Yichun	3.70	1.63	0.93	4.97	0.76
	Shanshi	4.42	1.37	0.85	3.10	0.36
	Mudanjiang	5.24	1.26	0.94	4.22	0.47
	Heihe	5.23	1.83	1.24	3.81	0.24
	Shangzhi	4.75	1.49	2.24	7.57	0.71
	Daxing’anling	4.03	1.65	0.70	4.67	0.32
	Elunchunqi	5.17	1.44	1.22	3.78	0.40

## 3 结论

实验将亲水相互作用色谱填料Xion作为吸附剂应用于五味子中木脂素类化合物的微萃取,研究建立了一种简单、高效的MSPD-HPLC方法萃取和测定五味子中5种木脂素类化合物。与经典的方法相比,所提出的MSPD具有样品用量少、有机溶剂消耗较低以及萃取时间短的优点。同时,该方法也为其他植物或药物制剂中木脂素类化合物的测定提供了参考。
